# High tumour burden, delayed diagnosis and history of cardiovascular disease may be associated with carcinoid heart disease

**DOI:** 10.3332/ecancer.2018.879

**Published:** 2018-10-25

**Authors:** Carolina Alves, Marcella Mesquita, Carolina Silva, Maria Soeiro, Ludhmila Hajjar, Rachel P Riechelmann

**Affiliations:** 1 Instituto do Câncer do Estado de São Paulo, University of São Paulo, São Paulo, Brazil; 2 AC Camargo Cancer Center, São Paulo, Brazil

**Keywords:** neuroendocrine tumours, carcinoid syndrome, carcinoid heart, metastases

## Abstract

**Background:**

Patients with carcinoid syndrome (CS) may present carcinoid heart disease (CHD) but prognostic factors are not entirely understood.

**Patients and Methods:**

Retrospective study of patients with metastatic neuroendocrine tumours (NETs) and CS and/or abnormal 24-hour-urinary 5-hydroxiindolacetic acid. CHD was defined as moderate to severe tricuspid or pulmonary regurgitation in the echocardiogram.

**Results:**

The frequency of CHD among 42 patients was 38% (95% confidence interval [CI]: 23%–54%). CHD was associated with higher volume of liver metastases (odds ratio [OR] 13.86, 95% CI: 2.57–74.68, *p* = 0.002). Time from CS symptoms to NET diagnosis was borderline significant (*p* = 0.08). When CHD was defined as at least mild tricuspide regurgitation, the frequency of CHD was 45% and it was associated with cardiovascular comorbidities (OR: 6.58, 95% CI: 1.09; 39.78, *p* = 0.040).

**Conclusion:**

CHD was frequent among patients with CS, significantly associated with high liver tumour burden, and likely linked to the history of cardiovascular disease and longer time of CS.

## Introduction

Neuroendocrine tumours (NETs) are rare malignancies derived from enterochromaffin cells that are distributed throughout the body. One of the most common functioning syndromes associated with NET is carcinoid syndrome (CS), caused by the secretion of bioactive amines, mainly serotonin, and characterised by facial flushing, diarrhea, dyspnea and bronchospasms [1]. The release of vasoactive substances produced by these tumours at the systemic circulation is associated with fibrotic complications, such as endocardial deposition of fibrotic plaques generally in the right-sided valve heart and retroperitoneal fibrosis [2, 3]. The fibrotic deposition in right-sided valves may lead to stenosis, chronic fibrotic degeneration that results in regurgitation and increased fluid pressure of the right atrium and ultimately, of the right ventricle, resulting in right-sided heart failure. This fibrotic heart valvular condition is called carcinoid heart disease (CHD). The presence of CHD is a serious condition because it is associated with high morbidity and mortality, with median survival times ranging from 2 years in the 1980s to 4.4 years in more recent reports [4, 5].

Since the bioactive amines have a pathogenic role, the use of therapies that control circulating serotonin levels (somatostatin analogues and antitumour drugs) could theoretically reduce the risk of developing CHD or prevent its progression. However, there is no evidence that treating CS delays the onset of valvular damage. When CHD is installed, surgical valve replacement is the only effective therapy, although associated with high morbi-mortality [5, 6]. Pharmacological and nonpharmacological treatments for right heart dysfunction, such as salt and water restrictions, diuretics and digitalis drugs, are used as symptomatic strategies. The diagnosis of CHD is made by an echocardiographic evaluation with Doppler [5, 6].

Previous studies have demonstrated that elevated (or abnormal) urinary 5-hydroxiindolacetic acid (5HIAA) and a number of daily flushing episodes are surrogate markers for the development and/or progression of CHD [7]. However, because CHD is relatively rare, more studies are needed to further explore why some patients develop CHD while others do not. For example, the frequency of CHD reported in the literature ranges from 20% to as high as 60% [3, 8]. Here, our objectives were to evaluate the frequency, characteristics and risk factors for CHD among patients with abnormal 5HIAA with or without CS treated in a large academic cancer centre.

## Methods

We performed a retrospective study of all consecutive adult patients with histologically confirmed advanced well-differentiated NET who were treated at Instituto do Cancer do Estado de São Paulo, Brazil, a large academic and public cancer centre. Patients were identified by an administrative list. Eligible patients had metastatic/inoperable NET of any origin with associated CS, which was defined as clinical symptoms of CS and abnormal (elevated) 24-hour-urinary 5-hydroxyindolacetic acid (5HIAA) or asymptomatic patients with abnormal 24-hour-urinary 5HIAA. Patients with midgut NETs are routinely screened for abnormal 24-hour-urinary 5HIAA at our institution regardless of CS [14]. All patients had performed echocardiograms to screen for CHD, following institutional routine. Since 2015, we have been screening all patients with abnormal 24-hour-urinary 5HIAA for CHD with echocardiograms.

In our institution, all patients with CS are treated with somatostatin analogues; upon progression, they may receive somatostatin analogue dose escalation, hepatic embolisation and everolimus or lutetium^177^, if there was a clinical trial available, since these therapies are not provided by the public system. The following clinical and demographic data were extracted from the electronic medical records: age at the time of diagnosis, sex, Eastern Cooperative Oncology Group *performance status* at the time of NET diagnosis, presence of cardiovascular comorbidities (defined as any cardiovascular condition that demanded the use of medications, e.g. arterial hypertension, coronary insufficiency, previous cardiac infarction), date of diagnosis (defined as the date of the biopsy), time from symptoms to the NET diagnosis (averaged in months), anatomical and pathological variables (tumour grade, Ki67 index, tumour stage, primary and metastatic sites and burden of hepatic metastases), 24-hour urinary levels of 5HIAA at the time of first echocardiogram (±1 month) and presence of known genetic syndrome. To quantify tumour burden, we retrospectively evaluated all digital images of abdominal computerised tomography or magnetic resonance performed at the time of the first echocardiogram and categorised liver involvement as absent, less than 50% or more than 50%. We also gathered information about treatments. To guarantee the accuracy of study data, all clinical, laboratory and radiological data were collected by two independent investigators and discrepancy was resolved by a third researcher.

To classify whether patients had CHD or not, we considered the first echocardiogram of those without CHD and the first that demonstrated signs of CHD for those patients with CHD. All echocardiograms were performed within a couple of months of the NET diagnosis and if no CHD was identified, they were repeated annually. All the digital videos of the echocardiograms were retrieved and reviewed by a single cardiologist experienced in the diagnosis of CHD to ensure accuracy and avoid misinterpretation. Given that there is no uniform definition of CHD, for this study, we defined CHD when there was echocardiographic evidence of moderate to severe tricuspid or pulmonary regurgitation; all other heart abnormalities were also sought and described in the Results section. Patients diagnosed with CHD were referred to the cardiologist for treatment decision.

Quantitative variables were represented by simple frequencies and percentages and qualitative variables by mean, standard deviation (SD), median, minimum, maximum and total of valid observations. Comparisons between patients with or without CHD and qualitative variables were made by the Chi-square test or, if necessary, Fisher’s exact test. Two-sided values of *P* < 0.05 were considered statistically significant. Univariate logistic regression analyses were conducted to explore factors associated with CHD. Variables at 25% level of significance in the univariate analyses were entered into a multivariate logistic regression model to evaluate independent factors linked to CHD.

## Results

From 2009 to 2017, 97 patients with advanced NET were identified with 42 patients fulfilling the criteria for CHD screening and being included in this study. Baseline characteristics are summarised in Table 1. Median age was 54.4 years (range, 19–85) and patients were more frequently female (57.1%). Midgut was the most frequent primary site (69%) and most primary tumours were G1 or G2 (95.2%). In a median follow time of 45.3 months, since the first echocardiogram, 16 patients (38% [95% confidence interval [CI]: 23%–54%) developed CHD. The median follow up times were 5.2 and 23.8 months among patients with and without CHD, respectively. Such difference occurred because many patients were diagnosed simultaneously with NET and CHD: eight patients presented CHD at the time of NET diagnosis and first echocardiograms. For those without CHD at diagnosis, three patients were found to have CHD 1 year after diagnosis of NET and five patients were diagnosed at 3, 6, 5, 8 and 9 years after the diagnosis of NET, with all CHD cases identified during routine echocardiogram screening.

Echocardiographic alterations found in patients with moderate to severe CHD (*N* = 16) were: pulmonary valve thickness in six patients (37.5%), pulmonary regurgitation in five patients (31.25%), tricuspid valve thickness in 11 (68.75%), tricuspid regurgitation in 16 (100%), right atrium dilatation in 12 (75%), right ventricular dilatation in nine patients (56.25%), right ventricular systolic dysfunction in one (6.25%), pericardial effusion in three (18.75%), accelerated influx in pulmonary artery in one (6.25%) and pulmonary arterial hypertension in ten patients (62.5%). Three patients had CHD without CS, with two presenting moderate tricuspid regurgitation and thickness and one, mild tricuspid reflux.

On univariate analyses (Table 2) no significant differences between patients with or without CHD were seen with respect to age (*p* = 0.79), sex (*p* = 0.38) or presence of bone metastases (*p* = 0.66). Flushing and/or diarrhea were more commonly observed in patients with CHD, although without statistical significance. The median level of 24-hour urine 5HIAA at diagnosis of CHD was numerically higher (1.6 times) among CHD patients (*p* = 0.20). CHD was significantly associated with the higher volume (50% or more of parenchyma involvement) of liver metastases (*p* = 0.002), and longer time from symptoms to diagnosis of NET (*p* = 0.08).

On multivariable analyses (Table 3), patients with higher tumour burden in the liver had a higher risk of developing CHD (OR: 13.86, 95 CI: 2.57–74.68, *p* = 0.002). Although abnormal 5HIAA and flushing were not significant in the univariate analyses, we still included them in the multivariable model because of their known association with CHD.

Because there is no standard definition for the diagnosis of CHD, we investigated risk factors for this condition, considering the presence of CHD when at least mild tricuspid regurgitation was identified by the echocardiogram. With this less conservative definition, CHD was observed in 19 patients (45%, 95% CI: 29%–61%). The new multivariable model (Table 4) found that higher liver involvement and presence of concurrent cardiovascular disease (OR: 6.58, 95% CI: 1.09; 39.78, *p* = 0.040) were associated with CHD; time from symptom to diagnosis was borderline significant.

Somatostatin analogues were the main treatment, being used by 88% of patients (Table 5), with 100% of patients with CS being treated with these agents. All CHD patients received clinical treatment, with pharmacological and nonpharmacological strategies; none had surgical treatment for CHD.

## Discussion

In this retrospective series of patients with NET and abnormally elevated 24-hour-urinary 5HIAA, we observed that CHD was frequent, with nearly 40% of them developing this heart complication during the course of their disease. We also found that high liver tumour burden, and possibly cardiovascular comorbidities and longer time from onset of tumour-related symptoms until the diagnosis of NET were associated with CHD. The 24-hour-urinary 5HIAA was also numerically higher in patients with CHD.

The exact underlying mechanisms that cause or induce CHD are unknown. While substances produced by NET are assumed to be the causative agents, the most likely factor associated with CHD development is serotonin. There are several preclinical and clinical studies pointing serotonin as the pivotal vasoactive NET-secreted hormone that triggers CHD. Preclinical studies have demonstrated that serotonin is a powerful mitogen for valvular subendocardial cells, leading to an increased valvular proliferation *in vitro* and *in vivo* [9, 10] and apparently through activation of 5-HT_2B_ receptors located in the heart [11]. On the clinical side, the levels of serotonin and 24-hour-urinary 5HIAA were significantly higher in patients who developed CHD versus those who did not [7, 12] and some serotonergic drugs such as ergot alkaloyds, cabergoline, *ecstasy* and fenfluramine have been reported to induce right-sided valvular fibrosis [13]. Although not significant, likely because of our small sample, the median levels of 24-hour-urinary 5HIAA were higher among patients with CHD. Likewise, high liver tumour burden was significantly linked to CHD, what reinforces the pathogenic effect of serotonin secretion into the post-hepatic circulation. However, serotonin alone does not explain why some patients with elevated 24-hour-urinary 5HIAA do not develop CHD. In our series, nearly 60% of patients did not present CHD after a median follow up of 45 months. While some may argue that this is a short period of time for CHD onset, 45 months represents the time from the first echocardiogram, not the time since the start of CS, which was approximately 19 months (1.6 years); we think the total time from initiation of CS of 64 months is a reasonable period to evaluate the development of CHD. The most likely explanation is that CHD is multifactorial. It is possible that other substances are co-secreted with serotonin. An interesting study showed that increased levels of activin A, a protein from the Transforming Growth Factor-β family, were found in the serum and endocardial plaques of patients with CHD [14]. Another argument against the exclusive role of serotonin in CHD is the lack of evidence that controlling CS and decreasing 24-hour-urinary 5HIAA levels with somatostatin analogues prevents the onset or progression of CHD [15]. Finally, another possibility is an underlying individual genetic susceptibility that could influence the development and progression of CHD or even other clinical factors, such as cardiovascular comorbid illnesses, which was identified as a risk factor in our study, when we classified CHD as at least any minor right-sided valvopathy.

Traditional cardiovascular risk factors in oncology have been described with certain chemotherapy agents [16] and tyrosine kinase inhibitors [17]. Specifically, in NET, a retrospective study of 71 NET patients who had performed a screening echocardiogram preoperatively or because of CS showed that patients who had received chemotherapy were significantly more likely to have CHD, with an odds ratio of 3.65 [15]. In our study, chemotherapy was used by only 6% of patients, what precludes any inferential analysis; actually, nowadays it would be harder to evaluate the role of chemotherapy as a risk factor for CHD because chemotherapy is not routinely recommended to treat advanced well-differentiated midgut NET [6]. To our knowledge, our study is the first that looked at and found that concurrent or previous cardiovascular disease (mostly arterial hypertension and coronary insufficiency) could be associated with CHD. While these findings have to be externally validated in larger study, they open new windows to examine the pathogenesis of CHD. Patients with previous cardiovascular comorbidities or cardiovascular risk factors often present endothelial dysfunction, marked by chronic endothelial inflammation and platelet aggregation dysfunction [18]. It is possible that CHD patients with comorbid cardiovascular illnesses have higher concentration of platelet serotonin in platelets located in the heart, leading to cardiac fibrosis through 5-TH_2B_ receptors. Corroborating with this hypothesis, an interesting pilot study evaluated 12 patients with and 10 without CHD who underwent cardiac catheterisation, aiming to measure and compare the serotonin plasma levels, collected from the femoral vein, and the platelets serotonin, collected from the right and left ventricles [19]. The investigators found that both levels of plasma and platelets serotonin were significantly higher among CHD patients, although they found no difference between right and left sides of the heart. On the other hand, it is also possible that our findings may simply reflect the low economic status of our patients who, being treated in the public setting, have less access to cardiology care. Larger studies need to confirm such findings.

The presence of flushing has been reported as a risk factor for CHD. In a prospective study, 250 patients with CS were followed with echocardiograms every 6 months, biochemical and radiological markers and clinical evaluations [7]. In a median follow up time of 29 months, 44 patients developed or progressed CHD (defined as at least 25% worsening the cardiac echocardiogram score), with three or more flushing episodes per day and 24-hour 5HIAA urinary level ≥ 300 μmol being identified as risk factors for CHD. In our study, probably due to our small sample and inaccuracy in quantifying a number of flushing in retrospective study, flushing was not a predictor of CHD.

In our study, patients with CHD had time from symptoms to NET diagnosis three times higher than patients without CHD. This finding suggests that longer exposure to circulating serotonin, higher liver tumour burden (also linked to higher serotonin levels) and/or uncontrolled CS may be linked to a higher risk of CHD. These finds are compatible with the literature, where CHD was more common among patients with a duration of 2.5–5 years to be diagnosed [4, 7]. It is highly probable that delayed diagnosis and treatment negatively affect CS patients, increasing the risk of CHD by longer exposure to vasoactive substances. In fact, our patients were treated in a public setting where the late diagnosis is common for many solid tumours. In our experience, CHD is rarely seen among NET patients treated in private hospitals, where there is a much faster access to diagnosis and initiation of cancer-directed therapies.

This study has some limitations. It was a single-centre, retrospective, uncontrolled study. Our results are limited by our small sample that prevented us to further explore CHD presence across subgroups of distinct cardiovascular diseases, and how different treatments influenced the progression of CHD. Also, an overall limitation is the lack of standardised definition for CHD. In our study, we decided to use more clinically relevant echocardiographic parameters to define CHD. On the other hand, strengths of our study are the inclusion of consecutive patients who performed echocardiogram to screen CHD, the review of all echocardiogram images by a single experienced cardiologist, the double collection of data from medical charts by two independent investigators to improve accuracy, and the availability of somatostatin analogues to treat CS.

Due to the rarity of CHD, some questions remain unanswered. While there is evidence that serotonin is involved in the pathogenesis of CHD, we do not know whether reducing its serum levels could prevent or delay the onset of CHD. Clinical trials with telotristat ethyl, an oral tryptophan hidroxylase inhibitor, which is an important enzyme for the synthesis of serotonin, may give us information about the outcomes of patients with CHD. The Telestar double-blind trial randomised patients with well-differentiated NET to receive either placebo or to two different doses (250 or 500 mg TID) of telotristate ethyl, with the objective to treat refractory CS, defined as at least four bowel movements a day, despite previous treatment with somatostatin analogues [20]. The trial was positive, with a significant reduction in the frequency of daily bowel movements, for both telotristat doses. Intriguingly, the preliminary data demonstrated that two CHD patients participating in Telestar had reduced cardiac fibrosis on serial echocardiograms [21].

## Conclusion

In this retrospective series, we found that patients with higher hepatic tumour burden were at a higher risk of CHD and that delayed diagnosis, concurrent cardiovascular disease (mostly arterial hypertension and coronary insufficiency) and higher 24-hour-urinary 5HIAA levels may also increase the risk of this condition. While CHD is a life-threatening complication of CS, there are few studies about its pathogenesis. Therefore, larger, multicentre and collaborative studies are urgently needed to better estimate the incidence of CHD, its risk factors and biology. With such information, clinical trials can be planned to improve the lives of patients with CHD. In addition, because we identified asymptomatic patients with CHD, we recommend that all patients with abnormal 24-hour-urinary 5HIAA be screened for CHD with an echocardiogram, regardless of CS.

## Conflicts of interest

The authors do not have any conflicts of interest to declare associated with this research.

## Funding statement

This research had no external funding.

## Figures and Tables

**Table 1.** Tumour characteristics by the presence of CHD.

Variables	With CHD [*N* = 16 (100%)]	Without CHD [*N* = 26 (100%)]
*Site*		
Midgut	10 (62.5)	19 (73)
Pancreas	1 (6.25)	1 (3.9)
Lung	1 (6.25)	2 (7.7)
Ovarian	2 (12.5)	0 (0)
Unknown	2 (12.5)	4 (15.4)
*Hereditary syndrome*		
MEN-1	1 (6.25)	0 (0)
No	15 (93.75)	26 (100)
*Functionality*		
Non-functioning	1 (6.25)	10 (38.4)
Functioning	15 (93.75)	16 (61.6)
CS	14 (93.3)	16 (100)
CS and gastrinoma	1 (6.45)	0 (0)
*Grade (OMS)*		
1	9 (56.25)	14 (53.9)
2	6 (37.5)	12 (44.1)
3	1 (6.25)	0 (0)
*Differentiation*		
Well	15 (93.75)	25 (96.2)
Poorly	1 (6.25)	1 (3.8)
*Ki67*		
Median	2% (1–60)	2% (1–15)

**Table 2.** Association between CHD and clinical and laboratory variables.

Variables	With CHD [*N* = 16 (100%)]	Without CHD [*N* = 26 (100%)]	Total [*N* = 42]	*p* value
*Sex*				
Female	11 (68.8)	13 (50)	24 (57.1)	0.384*
Male	5 (31.3)	13 (50)	18 (42.9)	
*Age at first echocardiogram*				
Median	61.7 (18.9–85.3)	52.3 (35.6–79.5)	54.4 (18.9–85.3)	0.790^#^
*Time from symptoms to NET diagnosis (years)*				
Mean (SD)	2.3 (2.6)	1.1 (1.9)	1.6 (2.2)	0.081^#^
*Liver metastases*				
< 50% or no parenchyma involvement	5 (31.3)	22 (84.6)	27 (64.3)	0.002*
≥ 50% of parenchyma	11 (68.8)	4 (15.4)	15 (35.7)	
No	1 (6.3)	3 (11.5)	4 (9.5)	
*Bone metastases*				
Yes	3 (18.8)	3 (11.5)	6 (14.3)	0.658**
No	13 (81.3)	23 (88.5)	36 (85.7)	
*Diarrhea*				
Yes	11 (68.8)	14 (53.8)	25 (59.5)	0.527*
No	5 (31.3)	12 (46.2)	17 (40.5)	
*Flushing*				
Yes	13 (81.3)	14 (53.8)	27 (64.3)	0.142*
No	3 (18.8)	12 (46.2)	15 (35.7)	
*Cardiovascular comorbidities*				
No	4 (25)	12 (46.2)	16 (38.1)	0.297*
Yes	12 (75)	14 (53.8)	26 (61.9)	
*24-hour 5HIAA urinary levels at first echocardiogram*				
Mean (standard deviation)	63.1 (44.3)	39.3 (64.6)	48.4 (58.3)	0.202^#^
Median (Minimum–Maximum)	52.4 (5.5–191)	14.8 (2.3–281)	26.9 (2.3–281)	

* χ^2^ test;

** Fisher’s exact test;

*** Likelihood ratio;

# T-Student test;

## Mann-Whitney test

**Table 3.** Multivariate logistic regression analysis of factors associated with CHD.

Variables	OR (95% CI)	*p* value
Time from symptoms to NET diagnosis (years)	1.35 (0.93; 1.96)	0.112
Liver metastases (grouped ≥ 50% versus < 50% or no)	13.86 (2.57; 74.68)	**0.002**
Presence of flushing	1.58 (0.24; 10.23)	0.634
24-hour 5HIAA urinary levels at first echocardiogram	1 (0.99; 1.01)	0.83

**Table 4.** Multivariate logistic regression analysis of factors associated with CHD defined as at least mild tricuspid regurgitation.

Variables	OR (95% CI)	*p*-valour
Time from symptoms to diagnosis (years)	1.42 (0.97; 2.08)	0.068
Liver involvement (≥ 50% versus < 50%/no)	8.63 (1.61; 46.28)	**0.012**
Presence of flushing	1.25 (0.22; 7.06)	0.797
Presence of cardiovascular disease	6.58 (1.09; 39.78)	**0.040**

**Table 5.** Treatments.

Variables	With CHD [*N* = 16 (100%)]	Without CHD [*N* = 26 (100%)]
Somatostatin analogues	16 (100)	21 (80.76)
Chemotherapy	1 (6.25)	2 (7.69)
Interferon	3 (18.75)	4 (15.38)
Embolisation	7 (43.75)	6 (23.07)
Lutetium ^177^	2 (12.5)	6 (23.07)
Everolimus	1 (6.25)	3 (11.53)
Metformin	1 (6.25)	4 (15.38)
